# Pulsar Emissions, Signal Modeling and Passive ISAR Imaging

**DOI:** 10.3390/s19153344

**Published:** 2019-07-30

**Authors:** Andon Lazarov

**Affiliations:** Department of Information Technologies, Naval Academy, 9026 Varna, Bulgaria; lazarov@bfu.bg; Tel.: +359-887-262-478

**Keywords:** passive ISAR asteroid imaging, pulsar emission signal modeling

## Abstract

The present work addresses pulsar Crab Nebula emissions from point of view of their modeling and applications for asteroid detection and imaging by applying inverse synthetic aperture radar (ISAR) principles. A huge value of the plasma’s effective temperature is a reason for pulsar emission coherency, a property of great practical meaning for a space objects navigation, localization and imaging. Based on measurement data obtained by Goldstone-Apple Valley and Arecibo radio telescopes, an original time frequency grid mathematical model of pulsar emissions is created. Passive ISAR scenario, a space object’s geometry and a model of pulsar signals reflected from the space object’s surface are also described and graphically illustrated. A new range compression approach for ISAR imaging is suggested and demonstrated. In order to reduce the level of additive white Gaussian noise in signals and enlarge the signal to noise ratio in the final image, coherent summation of multiple complex images is applied. To prove the correctness of the geometry, signal models and theoretical analysis, results of numerical experiments are provided.

## 1. Introduction

The pulsars are rotating neutron stars formed due to the collapse of massive stars core. They are the densest form of matter in the Universe. During the collapse stage the preservation of angular momentum causes the star to “spin-up” to a rotation period of order 10ms, whereas the preservation of magnetic flux drives the magnetic field strength at the stellar surface up to 10^12^ gauss or higher, with magnetic moments up to 10^26^ gaus-m^3^. Typical dimensions (radiuses) of white dwarfs which pulsate with period τ = 1 s, and neutron stars with pulsation period 10^−2^ s are in the interval $(1-5)\times 10^{6}$ m [1].

The pulsar can be considered as a massive freely spinning top and a powerful particle accelerator since the rotating magnetic field generates enormous electric fields that accelerate charged particles. These accelerated particles emit electromagnetic waves at spin frequency (across the spectrum, from radio waves to gamma-rays). Due to their enormous mass and relatively simple structure, pulsars are exceptionally stable rotators whose timing stability rivals that of conventional atomic clocks. It is the reason pulsar emissions to be used for spacecrafts radio navigation, asteroids detection, and imaging [2]. A navigation system based on celestial sources will be an independent positioning system and available in any Earth orbit as well as in interplanetary and interstellar space [3,4,5]. The stellar navigation accuracy depends on the emission’s time period, which for pulsars is with the high stability and accuracy.

Pulsar navigation tracking system based on the Doppler frequency measurement model and pulsar timing and an interplanetary navigation and positioning system using pulsar signals is discussed in [6,7]. The pulsar signal processing algorithm that consists of epoch-folding, matched filtering and detection is presented and evaluated in [8]. A hybrid detection algorithm based on energy and entropy analysis as an approach for spectrum sensing is considered in [9]. Principles of the pulsar navigation in the solar system are described in [10].

Pulsars are located on thousands of light years from Earth and can be considered as uninterruptible sources of electromagnetic waves. It allows the object illuminated by the pulsar beam as an asteroid or spacecraft, to be considered as a secondary emitter in compliance with Babinet’s principle. The diffracted electromagnetic field is a superposition of partial fields reemitted by all illuminated parts of the asteroid and carries information concerning its geometry and kinematics as a moving rigid body. The asteroid has an average orbital speed of 25 km/s. However, asteroids orbiting closer to a sun will move faster than asteroids orbiting between Mars and Jupiter and beyond. The average orbital speed of a main-belt asteroid is 17.9 km/s, the orbital speed of Ceres. If the asteroid comes from far away (e.g., the Oort Cloud), it will be accelerated by the Sun and achieve a velocity equal to the escape velocity from the Sun at the location of Earth, which is 30 km/s. The orbital speed and the escape velocity differ by a factor of 2^0.5^. That is, an object falling from infinity toward the Sun, will have a speed equal to 30 × 2^0.5^ = 42 km/s. Then the asteroid’s speed in the solar system varies from 17.9 km/s to 42 km/s.

In order to analyze and realize localization, navigation and imaging properties of pulsar emissions, adequate mathematical models need to be built, which is one of the goals of the present research. From point of view of pulsar emissions utilization, including localization, navigation and imaging, the most appropriate emissions are those from Crab Nebula pulsar. In this sense, the attention of the present work is on the signal modeling of the pulsar Crab Nebula emission and its passive inverse synthetic aperture radar application for asteroid imaging. The passive ISAR scenario, signal structure, power budget, image reconstruction and numerical experimental results are also discussed.

The remainder of the paper is organized as follows. In Section 2, a general description of Crab Nebula pulsar emission based on radio telescope measurements is given. In Section 3, an overview of synthetic and inverse synthetic aperture radar issues is suggested. In Section 4, passive ISAR scenario and asteroid’s geometry are described. In Section 5, a pulsar signal model as a time frequency grid and an algorithm of ISAR signal formation are described. In Section 6, a power budget, ISAR signal processing and asteroid image reconstruction are analytically described. In Section 7, results of a numerical experiment are provided and discussed. In Section 8, conclusion remarks are drawn.

## 2. Crab Nebula Pulsar Emission Based on Radio Telescope Measurements

It is known that powerful celestial sources with small angular diameters as quasars, sources of hydroxyl radical (OH)-emission and pulsars, are characterized by huge values of effective temperature, the main preposition for a coherent radiation mechanism [1]. The relativistic, magnetized plasma of pulsars radiates energy of (10^36^–10^38^)/*k*_B_, where *k*_B_ is the Boltzmann constant. This enormous value can be achieved only based on the coherency, the most important feature of pulsar electromagnetic emissions.

There exist measurement data obtained by radio telescopes that can be used for signal modeling purposes [11,12]. For instance, the mean profiles from the Crab pulsar consist of two frequency-dependent components, the main pulse (MP) and inter-pulse (IP). They appear at 70 and 215 degrees of the pulsar’s rotation phase, and can be identified from low radio frequencies to hard X-rays. The Main Pulses of the pulsar Crab emission are of particular interest for localization, navigation and imaging purposes. It is due to the coherency and stability of the Main Pulse’s repetition period with time duration 0.033 s.

The results of the observation show that most giant main pulses consist of several microbursts. The integrated intensity of pulsar flux is measured with a time resolution of 6.4 ns, whereas the dynamic spectrum is plotted with 19.5 MHz spectral resolution and 25.6 ns time resolution. It is worth noting that pulsar Crab emissions observed by Arecibo radio telescope are preliminary and coherently de-dispersed.

**Figure 1:**
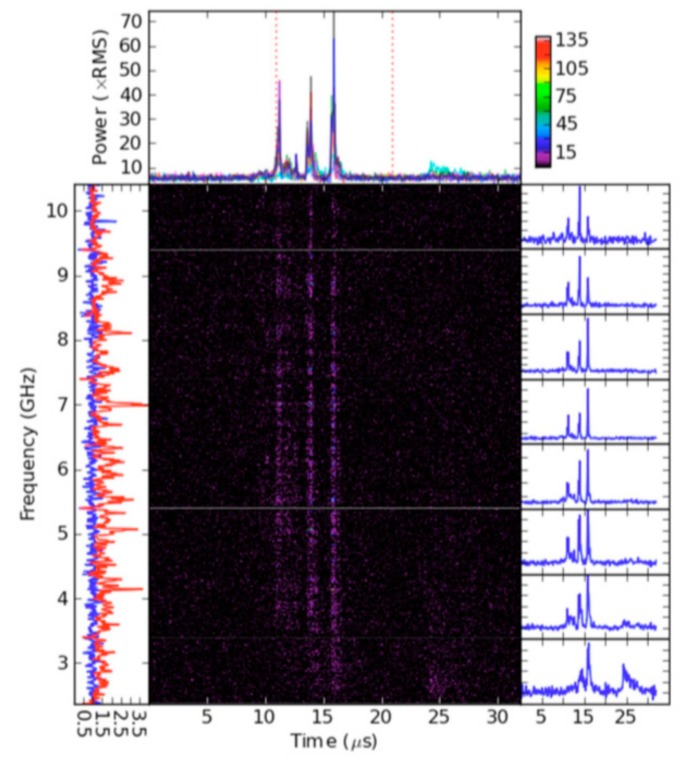
Typical main pulse time-frequency structure of Crab Nebula’s pulsar emission registered in Goldstone-Apple Valley radio telescope [12].

In Figure 1a typical main pulse with three microbursts, registered between 2 and 10 GHz with 125 ns time resolution and 8 MHz frequency spectrum resolution using the Goldstone-Apple Valley radio telescope is presented. The bar at the top right illustrates the generalized, over all frequency channels, root mean square (RMS) power level in the time-frequency coordinates. The left panel shows on-pulse (red) and off-pulse (blue) power as a function of frequency, integrated in time across the pulse. The high spikes in the on-pulse power correspond to strong, narrowband spikes within each microburst. On the right panel the pulse shape at eight frequencies within the pulse is depicted. The selected eight pulses are over-plotted in the top figure, where the dotted lines separate the on-pulse and off-pulse regions used for the left panel. All powers are given in terms of off-pulse noise (RMS) [12].

Based on characteristics of the main pulse defined in [5,11,12], it is worth noting that the main pulse consists of several microbursts, the time dimension of which is ≤1 µs long at 8−10 GHz, with a bandwidth ≥2 GHz. From measurement data depicted in Figure 1, the following inferences regarding the structure of the pulsar emission can be made.

1. In frequency channels from 4 to 9 GHz, the structure of the main pulse is preserved. The main pulse consists of microbursts almost with the same form. In each frequency channel three monochromatic microbursts can be revealed, which can be described as Gaussian pulses with different amplitudes.
2. The entire time-frequency record of the pulsar’s main pulse can be presented as a wideband signal. Each frequency component preserves time delay and, respectively, phase components induced by the object crossing the pulsar emission beam. The algebraic sum of all monochromatic components in frequency channels determines a sinc function with maximum in the position of a particular reemitting scattering point of the space object.

## 3. Synthetic and Inverse Synthetic Aperture Radar Issues

Problems of synthetic aperture radar imaging are widely discussed in the recent literature. Different modeling and imaging techniques including dominant scattering points and facets models, Fourier transforms, and phase compensation methods are in the focus of the authors’ attention. An adaptive ISAR imaging of maneuvering objects based on a modified Fourier transform and distributed ISAR sub-image fusion of a nonuniform rotating target based on matching Fourier transform are discussed in [13,14]. To mitigate blurring and defocusing effects induced by maneuvering targets on the process of ISAR imaging an original method based on modified chirp Fourier transform is suggested in [15]. A Fourier-based image formation algorithm for GNSS-based bistatic forward-looking synthetic aperture radar is presented in [16]. Returns form objects with complex motion in an ISAR imaging system are modeled as multicomponent quadratic frequency modulation (QFM) signals. QFM signals’ parameter estimation based on two-dimensional product modified parameterized chirp rate-quadratic chirp rate distribution is discussed in [17].

Non trivial ISAR image reconstruction methods are developed. An accurate method to extract ISAR images of multiple targets by applying Hough transform and particle swarm optimization (PSO) to find residual high order coefficients and to achieve better quality of ISAR imaging and moving target separation is discussed in [18]. A method including a genetic algorithm, PSO and PSO with an island model, to compensate for the inter-pulse phase errors caused by the target movement in stepped-frequency ISAR imaging is proposed in [19]. Residual motion error correction with back-projection multi-squint algorithm for airborne synthetic aperture radar interferometry is applied in [20].

An object’s movement of higher order as different kinds of accelerations and range displacements causes the ISAR image to be blurred, which requires focusing the object. An autofocusing method for improving synthetic aperture radar image quality and modified fractal signature image classification technique are described in [21]. An algorithm based on keystone transform and time-domain chirp scaling to deal with the space-variant range cell migration in ISAR imaging with ultrahigh range resolution is proposed in [22]. Phase compensation and image autofocusing algorithms using randomized stepped frequency emitted ISAR signals are described in [23].

A problem of coherent integration for detecting high-speed maneuvering targets, involving range migration, quadratic range migration, and Doppler frequency migration within the coherent processing interval, and a coherent integration algorithm based on the frequency-domain second-order phase difference approach are discussed in [24]. Multi-sensor ISAR radar imaging and phase adjustment based on a combination of signal sparsity and total variation is considered in [25].

To improve the azimuth resolution of ISAR images a fractional sparse energy representation method combined with fractional Fourier transform is proposed in [26]. The clock jitter influence on the signal to noise ratio (SNR) of an analog-to-digital-converter of the ISAR signal acquired from the space object is analyzed in [27].

The abilities of the astronomical radars as unique and powerful information tools to measure physical properties and orbital parameters of asteroids are thoroughly analyzed and illustrated in [28].

Based on the aforementioned, the present work will focus on the passive ISAR scenario—kinematics and geometry—as well as signal modeling and special solutions in the asteroid’s imaging algorithm.

## 4. Passive ISAR Scenario and Asteroid Kinematics and Geometry

**Figure 2:**
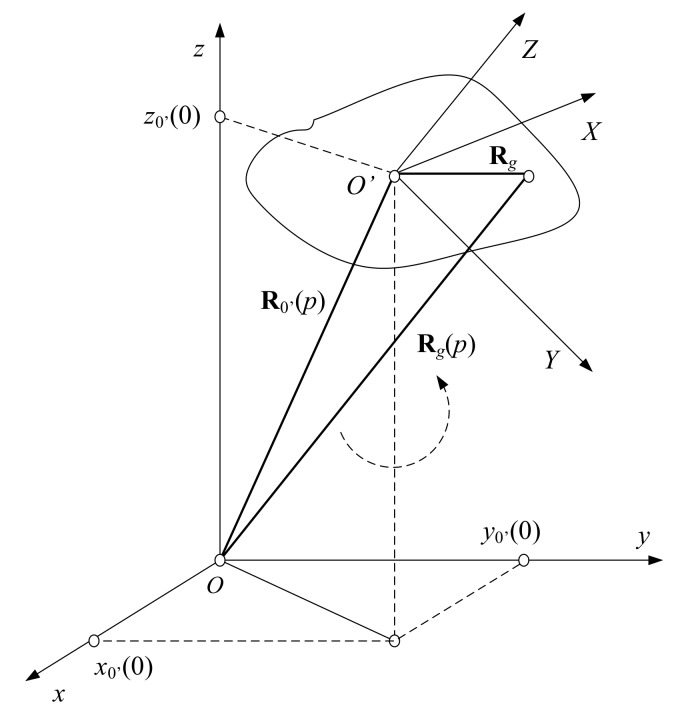
Inverse synthetic aperture radar (ISAR) scenario: asteroid’s kinematics and geometry.

Consider a 3D regular grid, where the asteroid’s geometry is described. The grid is defined in Cartesian system O′XYZ and moves on a rectilinear trajectory at a constant speed in the coordinate system Oxyz. The mass-center of the object, the geometric center of the 3D grid and the origin of the coordinate system O′XYZ coincide in point *O*’ Figure 2. The distance vector from the radio telescope placed in the origin of the 3D observation coordinate system Oxyz to the *g*-th generic point of the object space, measured at the *p*-th moment is described by the vector equation

(1) $$R_{g}(p)=R_{0\prime }(p)+AR_{g}$$

where $R_{0\prime }(p)=R_{0\prime }(0)+VT_{p}\cdot p$ is the mass center position vector; $R_{g}(p)={\left[x_{g}(p),y_{g}(p),z_{g}(p)\right]}^{T}$ is the generic point’s distance vector; $x_{g}(p),y_{g}(p)$, and $z_{g}(p)$ are the current coordinates of the generic point, $p=\bar{0,\;N-1}$; N the number and full number of emitted pulses, respectively, during time of observation (aperture synthesis); $R_{0\prime }(0)={\left[x_{0\prime }(0),y_{0\prime }(0),z_{0\prime }(0)\right]}^{T}$ is the line-of-sight vector of the object’s geometric center at the moment p=0; $V={\left[V\cos\alpha ,V\cos\beta ,V\cos\delta \right]}^{T}$ is the object’s linear vector velocity; $R_{g}={\left[X_{g},Y_{g},Z_{g}\right]}^{T}$ is the distance vector to the *g*-th generic point in the coordinate system O′XYZ; $X_{g}=g_{X}\cdot (\Delta X)$, $Y_{g}=g_{Y}\cdot (\Delta Y)$, and $Z_{g}=g_{Z}\cdot (\Delta Z)$ are the discrete coordinates of the *g*-th generic point in the coordinate system O′XYZ; $g_{X}$, $g_{Y}$, $g_{Z}$ are the coordinate indexes of the generic point; ΔX, ΔY, and ΔZ are the spatial dimensions of the 3D grid cell; cosα, cosβ, and $\cos\gamma =\mathrm{mod}\left(\sqrt{1-\cos^{2}\alpha -\cos^{2}\beta }\right)$ are the guiding cosines; and *V* is the module of the linear velocity vector. The elements of transformation-rotation matrix A are defined by Euler’s expressions

(2) $$\begin{array}{l} a_{11}=\cos\psi \cos\varphi -\sin\psi \cos\theta \sin\varphi ;\\ a_{12}=\cos\psi \sin\varphi +\sin\psi \cos\theta \cos\varphi ;\\ a_{13}=\sin\psi \sin\theta ;\\ a_{21}=-\sin\psi \cos\varphi -\cos\psi \cos\theta \sin\varphi ;\\ a_{22}=-\sin\psi \sin\varphi +\cos\psi \cos\theta \cos\varphi ;\\ a_{23}=\cos\psi \sin\theta ;\\ a_{31}=\sin\theta \sin\varphi ;\\ a_{32}=-\sin\theta \cos\varphi ;\\ a_{33}=\cos\theta \cdot \end{array}$$

where in case of a rotating object, ψ, φ, and θ are time dependent angles, yaw, pitch and roll, respectively.

## 5. Pulsar Signal Formation

Models of pulsars’ signals can be created based on the structure of their continuous electromagnetic emissions. The pulsar signal detected by a radio telescope is registered in multiple frequency channels Figure 1 as a set of sinusoids (waveforms) having almost the same Gaussian envelopes [5,8,11,12]. This allows the pulsar signal model to be described as a time-frequency grid.

### 5.1. Time-Frequency Grid Pulsar Signal Model

For each frequency channel the pulsar signal model can be expressed as

(3) $$s_{r}(t)=\sum_{p=0}^{N-1}\sum_{l=0}^{L-1}a_{l}\cdot \exp\left[-\frac{{\left(t-T_{pl}\right)}^{2}}{2\sigma_{p}^{2}}\right]\cdot \exp\left[j.2\pi \cdot f_{r}\cdot (t-T_{pl})\right]$$

where $f_{r}$ is the frequency of the r-th frequency channel, defined by the expression $f_{r}=f_{0}+r\cdot \Delta f$, where $r=\bar{0,\;R-1}$ is the frequency channel’s number, *R* is the number of the frequency channels the signal is registered in, $f_{0}$ is the frequency of the 0-th channel, Δf is the difference between frequency channels (spectral resolution), $a_{l}$ is the amplitude of the Gaussian microburst, $\sigma_{l}$ is the time width of the *l*-th Gaussian microburst, $l=\bar{0,\;L-1}$ is the index of the Gaussian microburst, L is the number of microbursts inside the main Pulse,p is the index of the main Pulse or the index of the Main Pulse’s repetition period, $T_{pl}$ is the composed repetition period defined by $T_{pl}=p\cdot T_{p}+l\cdot T_{l}$, where $T_{p}$ is the main pulse repetition period, $T_{i}$ is the Gaussian microburst repetition period inside the main pulse, *t* is the current time, which in discrete form can be presented as $t=T_{pl}+(k-1)\cdot \Delta T$, where *k* is the time sample index inside the microburst, ΔT is the sample’s time duration inside the microburst.

**Figure 3:**
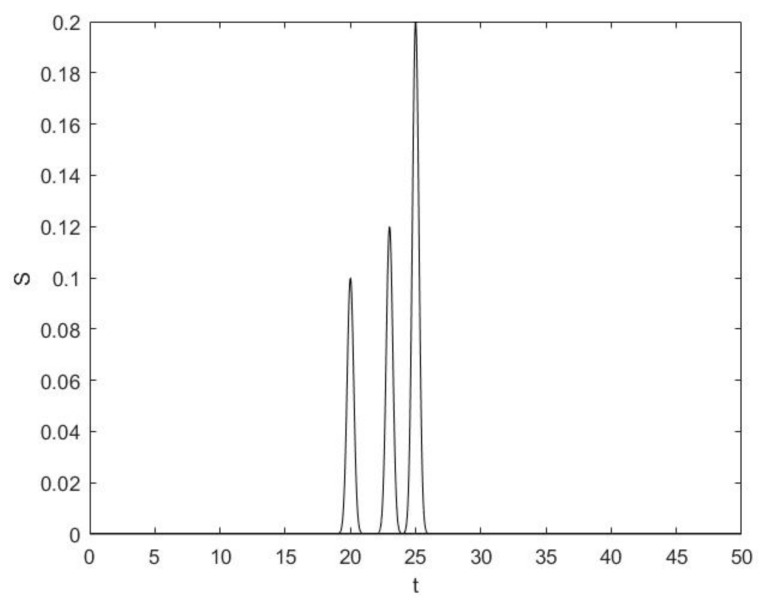
Main pulse with three microbursts measured in a particular frequency channel.

A generalized model of the main pulse with three microbursts with Gaussian envelopes registered in a particular frequency channel is presented in Figure 3.

### 5.2. A Model of a Pulsar Signal Reflected from a Space Object

Signals reflected from the space object preserves the frequency characteristics of the pulsar emission except the amplitude and time delay from a particular generic point of the object surface or from the entire object. The signal amplitudes can be instrumentally measured whereas the time delay can be defined by the correlation of the received real signal (signal plus additive Gaussian noise) reemitted by a pseudo stationary object, slow moving in short integration time interval, with a priory known pulsar signal with time displacement. The signal reemitted by a particular generic point of the asteroid or from the entire surface is a delayed copy of a pulsar signal and can be interpreted as a time-frequency grid signal model with time delay. The pulsar’s main pulse train with *L* microbursts reemitted by the *g*-th asteroid’s generic point and registered in the *r*-th frequency channel is modeled by the expression

(4) $${\hat{s}}_{r,g}(t)=\sum_{p=0}^{N-1}\sum_{l=0}^{L-1}a_{g}\cdot \exp\left[-\frac{{\left(t-T_{pl}-t_{g}\right)}^{2}}{2\sigma_{l}^{2}}\right]\cdot \exp\left[j.2\pi \cdot f_{r}\cdot (t-T_{pl}-t_{g})\right],$$

where $a_{g}$ is the amplitude of the reemitted *l*-th microburst from the *g*-th generic point; $\sigma_{l}=T/2$ is the time dispersion of the Gaussian microburst; *T* is the microburst’s time width; $t_{g}=t_{g}(p)=R_{g}(p)/c$ is the time delay of a reemitted signal by the *g*-th generic point from the object’s surface; $R_{g}(p)={\left[x^{2}(p)+y^{2}(p)+z^{2}(p)\right]}^{\frac{1}{2}}$ is the current distance to the particular generic point from the object; *c* is the speed of light in vacuum.

The time delays $t_{g}(p)$ for p-th main pulse and *l*-th microburst are arranged in ascending order, i.e., *g* = 0, 1, 2, 3, …, *G*-1, where *G* is the full number of asteroid’s generic points, *g* = 0 is the index of the minimum time delay from the nearest generic point, i.e., $t_{0}(p)=t_{g,\min}(p)$, g = *G* -1 is the index of the maximum time delay from the furthest generic point, i.e., $t_{G-1}(p)=t_{g,\max}(p)$.

For the *p*-th main pulse and *l*-th microburst in the sequence of pulsar emissions the signal reemitted by *g*-th generic point limited by the microburst’s time width, *T*, can be rewritten as

(5) $$s_{r,g}(t)=a_{g}\cdot \mathrm{rect}\left(\frac{t-T_{pl}-t_{g}}{T}\right)\cdot \exp\left[-\frac{2\cdot {\left(t-T_{pl}-t_{g}\right)}^{2}}{T^{2}}\right]\times \exp\left[j\cdot 2\pi \cdot f_{r}\cdot (t-T_{pl}-t_{g})\right],$$

where $rect\left(\frac{t-T_{pl}-t_{g}}{T}\right)=\left\{\begin{array}{l} 1,if\;0\leq \frac{t-T_{pl}-t_{g}}{T}<1\\ 0,\;\mathrm{othewise} \end{array}\right.$.

The following substitutions are made $t=T_{pl}+t_{kp}$, $t_{kp}=t_{g,\min}(p)+k\cdot \Delta T$, ${\hat{t}}_{g}(p)=t_{kp}-t_{g}(p)$, where $k=\bar{0,\;K+(k_{\max}-k_{\min})-1}$ is the current signal sample’s number in the microburst and/or the signal sample’s number measured on the range direction, K=int(T/(ΔT) is the full range signal samples’ number, $k_{\max}=\mathrm{int}[t_{g,\max}/(\Delta T)]$ is the index of the range bin where the signal reemitted by the furthest generic point with time delay $t_{g,\max}$ is recorded, $k_{\min}=\mathrm{int}[t_{g,\min}/(\Delta T)]$ is the index of the range bin where the signal reemitted by the nearest generic point with time delay $t_{g,\min}$ is recorded, the difference $\left(k_{\max}-k_{\min}\right)$ denotes the relative object’s time width, measured on range direction. The analytical discrete model of the ISAR signal reemitted from the entire surface of the asteroid for each k,p, and r can be written as

(6) $$S(k,p,r)=\sum_{g\in G}a_{g}\cdot \mathrm{rect}\left(\frac{{\hat{t}}_{g}(p)}{T}\right)\cdot \exp\left[-\frac{2\cdot {\left({\hat{t}}_{g}(p)\right)}^{2}}{T^{2}}\right]\cdot \exp\left[j.2\pi \cdot f_{r}\cdot {\hat{t}}_{g}(p)\right]$$

where G is the asteroid’s object space.

### 5.3. ISAR Signal Modeling Algorithm

**Figure 4:**
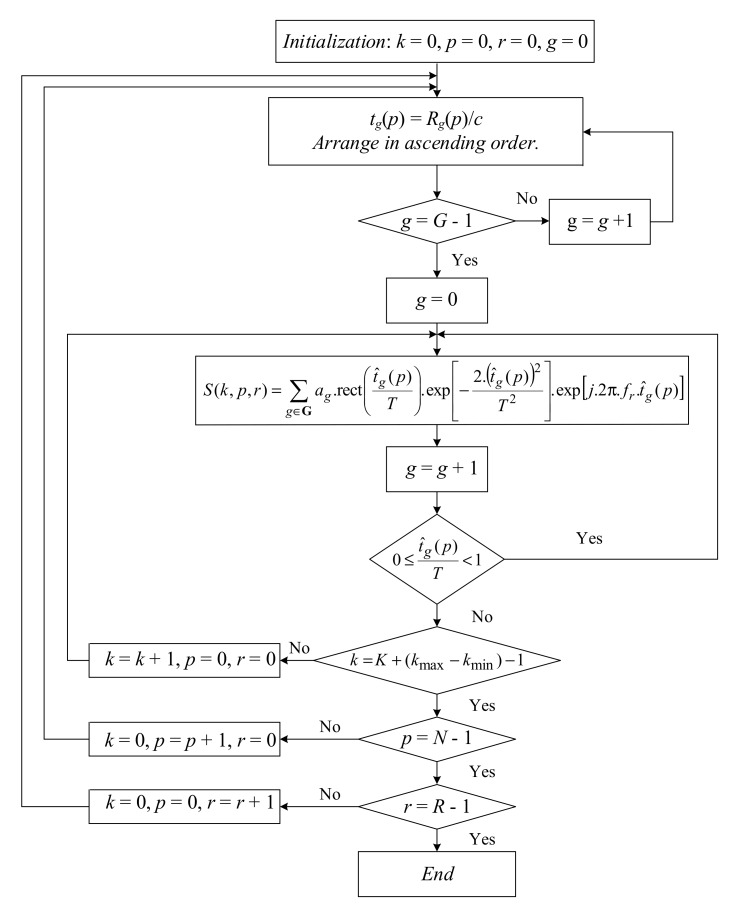
Flow chart of asteroid’s ISAR signal modelling.

The flow chart of asteroid’s ISAR signal modelling is presented in Figure 4. The algorithm consists of two parts. In the first part, calculations of time delays, $t_{g}(p)=R_{g}(p)/c$, for each g∈G, of signals reemitted by asteroid’s scattering points and their arrangement in ascending order, $g=\bar{0,\;G-1}$, are performed. In the second part, the asteroid’s ISAR signal modeling in accordance with the expression (7) is accomplished. The summation is correct if and only if the inequality $0\leq \frac{{\hat{t}}_{g}(p)}{T}<1$ holds, otherwise for particular r and p, k increases, and the procedure is repeated until $k=K+(k_{\max}-k_{\min})-1$, then for particular r and k, p increases, and the procedure is repeated until p=N−1, then for particular p and k, r increases, and the procedure is repeated until r=R−1, which is the end of the asteroid’s ISAR signal formation.

Only one microburst is considered. The ISAR signal microburst in the main pulse sequence, reemitted by the asteroid, after preliminary signal processing (signal detection and de-dispersion) is recorded in two-dimensional coordinates, time *t* and frequency $f_{r}$. The signal time record is divided into two coordinates: fast time, measured on the range direction with index *k* (range sample number), and slow time measured in cross-range direction with index *p* (azimuth sample number). Thus, the ISAR signal microburst is registered in a three-dimensional array with discrete coordinates [*k*, *p*, *r*]. In case all microbursts are used for aperture synthesis, the procedure is repeated for all remained microbursts inside the main pulse.

## 6. ISAR Signal Processing and Asteroid Image Reconstruction

### 6.1. Power Budget

Since the asteroid’s ISAR signal distinguishes with a low signal power density on the Earth’s surface, long observation times will essentially enhance the signal to noise ratio (SNR), which can be defined by the modified radar equation [29]

(7) $$\mathrm{SNR}=\frac{S\cdot \Delta F\cdot G\cdot \lambda^{2}\cdot \sigma \cdot T_{\mathrm{int}}\cdot n\cdot N}{4\cdot \pi^{2}{\left(R_{0\prime }\right)}^{2}k_{B}\cdot T_{s}}$$

where *S* is the spectral flux density of the asteroid’s signal on the Earth measured in $\left[\frac{W}{m^{2}\cdot \mathrm{Hz}}\right]$; $G=q{\left(\frac{\pi \cdot D_{pr}}{\lambda }\right)}^{2}$ is the parabolic reflector antenna gain; $D_{pr}$ is the diameter of the parabolic reflector; *q* is the efficiency factor which is around 0.5 to 0.6; $\sigma =\frac{\pi^{3}\cdot D^{4}}{4\cdot \lambda^{2}}$ is the asteroid’s radar cross section; $k_{B}=1.38\times 10^{-23}$ $\left[\frac{W}{\mathrm{Hz}\cdot K}\right]$ is the Boltzmann constant; *D* is the asteroid’s diameter; $R_{0\prime }$ is the distance to the asteroid’s mass center at the moment of imaging; $T_{s}=410\;K$ is the receiver noise temperature; $T_{\mathrm{int}}=T$ is the integration time equal to the microburst time width; *N* is the number of main pulses for aperture synthesis in one imaging segment; n is the number of imaging segments. In case the spectral flux density of the direct pulsar signal is $10^{-23}$$\left[\frac{W}{m^{2}\cdot \mathrm{Hz}}\right]$, assume the spectral flux density of the asteroid’s signal $S=10^{-26}$$\left[\frac{W}{m^{2}\cdot \mathrm{Hz}}\right]$. To evaluate SNR assume *N* = 128, $\Delta F=2\times 10^{9}$ Hz; $D_{pr}=300$ m; λ=0.03 m; *D* = 100 m; $T_{\mathrm{int}}=2\times 10^{-6}$ s; $R_{0\prime }=10^{9}$ m. In case $n=10^{2};\;10^{3};\;10^{4};\;10^{5};\;10^{6}$, the enhancement (reducing of negative values) of the SNR in dB with the number of imaging segments is presented in Table 1.

The negative value of SNR (−27 dB) can be further reduced by multiple coherent summations of the current complex image with the previous one after each accomplishment of the image reconstruction procedure.

### 6.2. Range Compression of the ISAR Signal

Considering that the ISAR signal reemitted by the asteroid is registered in a time-frequency grid, the range compression can be performed by the algebraic summation of signals from all frequency channels. To prove this statement the range compressing will be illustrated assuming that the frequency difference between channels, ∆*f* tends to zero. It allows discrete time and continuous frequency approach to be applied in the ISAR signal’s range compressing. The range compressed signal can be expressed as a frequency integration of a pulsar ISAR signal, i.e.,

(8) $$\hat{S}(k,p)=\int_{f_{c}-\frac{\Delta F}{2}}^{f_{c}+\frac{\Delta F}{2}}\sum_{g\in G}{\tilde{a}}_{g}[{\hat{t}}_{g}(p)]\cdot \exp\left[j.2\pi \cdot f\cdot {\hat{t}}_{g}(p)\right]df,$$

where ${\tilde{a}}_{g}[{\hat{t}}_{g}(p)]=a_{g}\cdot \mathrm{rect}\left(\frac{{\hat{t}}_{g}(p)}{T}\right)\exp\left[-\frac{2\cdot {\left({\hat{t}}_{g}(p)\right)}^{2}}{T^{2}}\right]$ is the time dependent amplitude*,*$f_{c}=f_{0}+(\Delta F/2)$ is the central channel frequency, ΔF=R·Δf is the frequency bandwidth.

After simple mathematical manipulations the solution of the integral can be written as

(9) $$\hat{S}(k,p)=\sum_{g\in G}{\tilde{a}}_{g}[t_{kp}-t_{g}(p)]\cdot \Delta F\cdot \exp\left[j2\pi [t_{kp}-t_{g}(p)]\cdot f_{c}\right]\cdot \frac{\sin\left(\pi [t_{kp}-t_{g}(p)]\cdot \Delta F\right)}{\pi [t_{kp}-t_{g}(p)]\cdot \Delta F}$$

**Figure 5:**
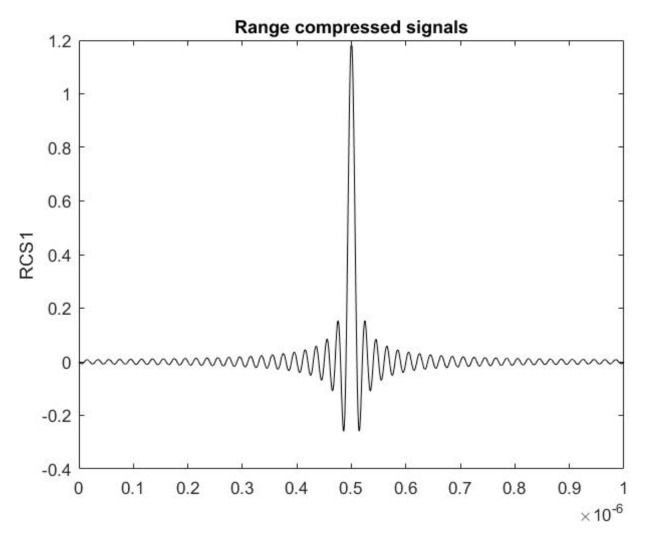
A range compressed signal RCS1 with time delay 0.5 μs.

Thus, the range compressed ISAR signal is a time displaced copy of a sinc function defining the position of *g*-th scattering point from the space object, the asteroid. In Figure 5, a range compressed signal with time delay *t*_1_ = 0.5  μs is presented.

**Figure 6:**
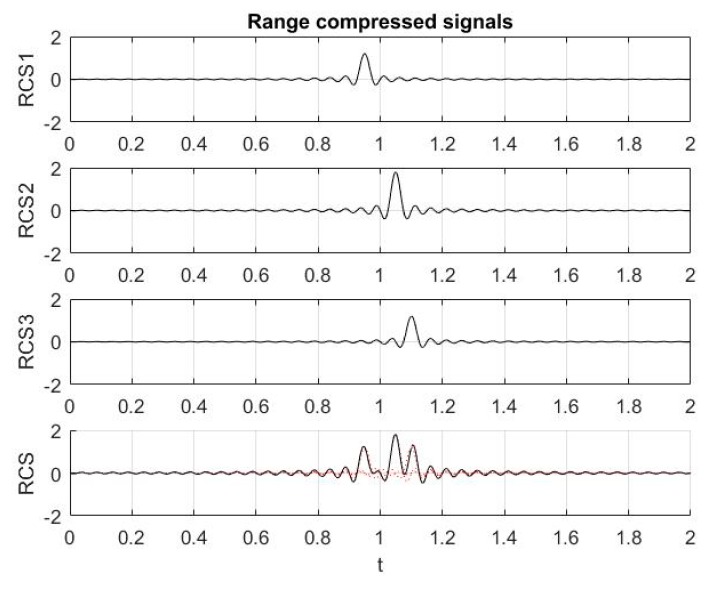
Three range compressed signals RCS1, RCS2, RCS3 and their sum RCS.

In Figure 6, three range compressed signals RCS1, RCS2, RCS3 and their sum RCS, is depicted with the following parameters: microburst time width 2 μs, frequency band width ∆*F* = 100 MHz, signals’ time delays: *t*_1_ = 0.5 μs, *t*_2_ = 0.515 μs, *t*_3_ = 0.52 μs, and amplitudes: $a_{1}=1.2$, $a_{2}=1.8$, $a_{3}=1.2$.

It is worth noting that in case ∆*f* differs from zero (i.e., has a finite value) an unambiguous time interval ∆τ of the ISAR compressed signal registration has to be defined (i.e., ∆τ = 1/∆*f*).

In a discrete time-frequency grid of the asteroid’s signal registration, the range compression can be expressed as

(10) $$\hat{S}(k,p)=\sum_{r=0}^{R-1}\sum_{g\in G}{\tilde{a}}_{g}[{\hat{t}}_{g}(p)]\cdot \exp\left(j.2\pi \cdot f_{r}\cdot {\hat{t}}_{g}(p)\right)$$

### 6.3. Azimuth Compression of the Range Compressed ISAR Signal and Complex Imaging

Pulsar ISAR signals reemitted by the asteroid are registered in a far field zone of electromagnetic waves propagation. It means that a plane wave approximation can be applied and, hence, an inverse Fourier transform can be used in order to perform azimuth compression of the range compressed ISAR signal, i.e.,

(11) $$\hat{S}(k,\hat{p})=\frac{1}{N}\sum_{p=0}^{N-1}\hat{S}(k,p)\cdot \exp\left(j\frac{2\pi \cdot p\cdot \hat{p}}{N}\right),$$

where $\hat{p}=\bar{0,\;N-1}$ is the discrete coordinate of the asteroid’s generic point at the moment of imaging.

The inverse Fourier transform (11) is a correlation procedure, searching for all Doppler components $\exp\left(-j\frac{2\pi \cdot p\cdot \hat{p}}{N}\right)$ inside the spatial spectrum $\hat{S}(k,\hat{p})$, i.e., searching for that $\hat{p}$ in the interval from 0 to (*N* – 1) that reveal amplitudes by maximizing their intensities $\mathrm{mod}\left[\hat{S}(k,\hat{p})\right]$, and compensate for all phases $\arg[\hat{S}(k,\hat{p})]$ induced by the radial velocities, the motion of first order, except phases proportional to the radial velocities (Doppler frequencies) of scattering points at the moment of imaging. The coordinate $\hat{p}$ is proportional to a constant radial velocity (Doppler frequency), that corresponds to the azimuth position of a particular scattering point at the moment of imaging. Thus, the expression (11) defines the asteroid’s complex image with amplitude $\mathrm{mod}[\hat{S}(k,\hat{p})]$, and phase $\arg[\hat{S}(k,\hat{p})]$. It is worth noting that the Doppler bandwidth and, respectively, Doppler resolution or cross range (azimuth) resolution of the ISAR signal are limited by the fixed main pulse repetition period equal to 0.033 s, and apparent rotation angle between the vector velocity and mass-center’s line-of-sight vector in case rectilinear movement of the asteroid.

## 7. Numerical Experiment

In order to validate the mathematical derivation of asteroid’s ISAR geometry, kinematics and signal models, a numerical experiment with a signal model of one microburst is carried out based on the following pulsar’s signal parameters: central channel frequency $f_{c}$ = 10.075 GHz; frequency channels bandwidth ∆*F* = 150 MHz; spectral resolution ∆*f* = 1MHz; $f_{\min}=10$ GHz,$f_{\max}=10.15$ GHz; number of frequency channels *R* = 150; main pulses time repetition period $T_{p}=0.033$ s, microburst time width $T=10^{-6}$ s; number of range samples in microburst K=128; sample’s time width $\Delta T=0.8\times 10^{-8}$ s; number of azimuth measurements *N* = 128 in one imaging segment defined by the number of main pulses used for aperture synthesis; initial coordinates of asteroid’s detection *x*_0′_ = 1 km, y_0′_ = 350 km, z_0′_ = 10^3^ km; distance to the mass-center $R_{0\prime }=1.06\times 10^{6}$ km; asteroid’s velocity V=35 km/s; velocity angles α = π/6, β = π/4, γ = π/2. The geometry of the asteroid is depicted in a 3-D grid with dimensions 64 × 64 × 64, and grid cell dimensions ∆X = ∆Y = ∆Z = 0.5 m, Relative intensity of scattering points is $a_{g}=10^{-3}$. In order to obtain an asteroid’s image of high resolution the apparent rotation angle between the asteroid’s vector velocity and mass-center’s line-of-sight vector has to be no less than 10^0^. It guaranties the Doppler displacement in the spectrum of the ISAR signal to realize the necessary azimuth resolution of the asteroid’s image.

Considering that the ISAR signal from the asteroid is very weak in comparison with the thermal noise, the experiment is carried out assuming the signal is obscured by additive white Gaussian noise, an appropriate model of signal disturbances in deep space navigations and communications. Assume as follows: number of imaging segments *n* = 100, $S=10^{-26}$$\left[\frac{W}{m^{2}\cdot \mathrm{Hz}}\right]$, $\Delta F=0.15\times 10^{9}$ Hz, $D_{pr}=300$ m, λ=0.03 m, asteroid’s diameter *D* = 29 m, $T_{\mathrm{int}}=10^{-6}$ s, then the signal to noise ratio calculated by (7) is equal to −15 dB. To reduce the level of noise, coherent summation of multiple complex ISAR images obtained after multiple applications of the image reconstruction procedure is applied. The number of complex images’ sums mitigating the level of the additive white Gaussian noise is 10.

**Figure 7:**
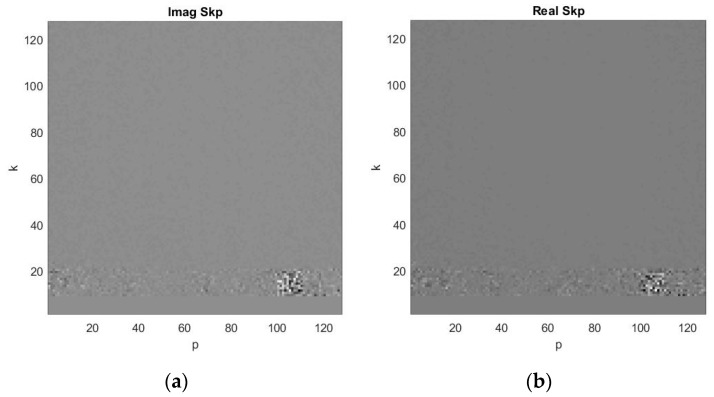
Range compressed ISAR signal obtained after summation of signals in all frequency channels: (**a**) real part; (**b**) imaginary part.

The ISAR signal from the asteroid is modelled in accordance with the algorithm presented by the flow chart in Figure 4. In each frequency channel additive white Gaussian noise is added to the signal using a standard procedure. The complex range compressed ISAR signal is modelled by expression (10). The real and imaginary parts of the range compressed ISAR signal obtained after summation of the signals from 150 frequency channels and registration in range (*k*) and azimuth (*p*) coordinates are depicted in Figure 7a,b respectively.

**Figure 8:**
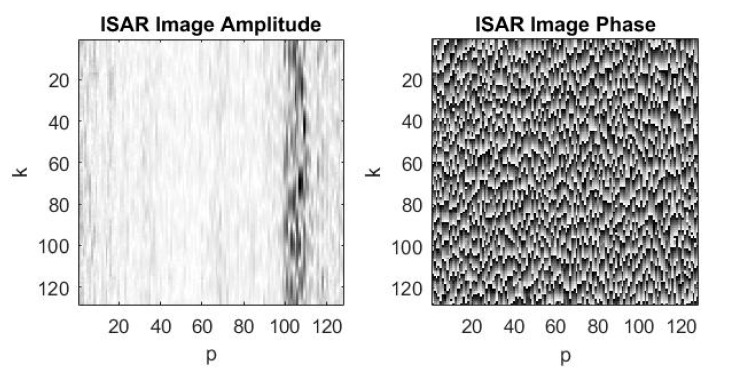
Complex image after azimuth compression of the range compressed ISAR signal with –15 dB signal to noise ratio: (**a**) ISAR image amplitude; (**b**) ISAR image phase.

The ISAR complex image as ISAR amplitude and ISAR phase is extracted from the range compressed signal by applying azimuth compression with inverse Fourier transform (11) realized by inverse fast Fourier transform. The ISAR image amplitude and the ISAR image phase, the complex image with (−15) dB signal to noise ratio, just after azimuth compression of the range compressed ISAR signal, are presented in Figure 8a,b, respectively. The asteroid’s image is obscured by noise.

**Figure 9:**
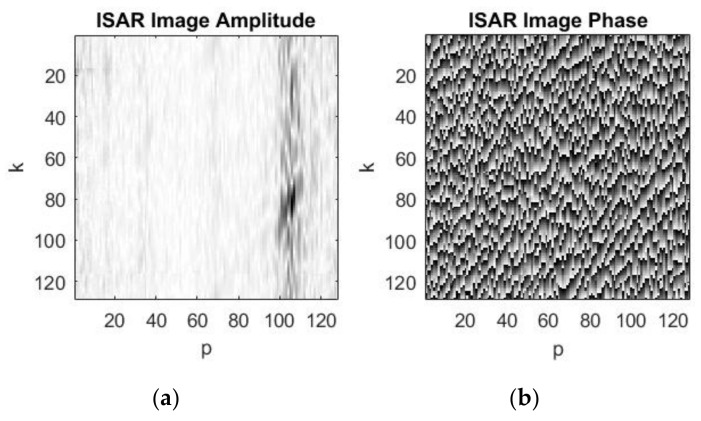
Resulting complex image after 3rd additive summation of complex images: (**a**) ISAR image amplitude; (**b**) ISAR image phase.

**Figure 10:**
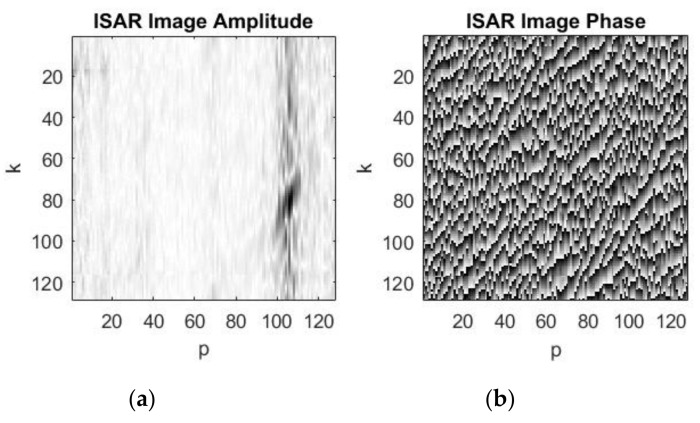
Resulting complex image after 8th additive summation of complex images: (**a**) ISAR image amplitude; (**b**) ISAR image phase.

**Figure 11:**
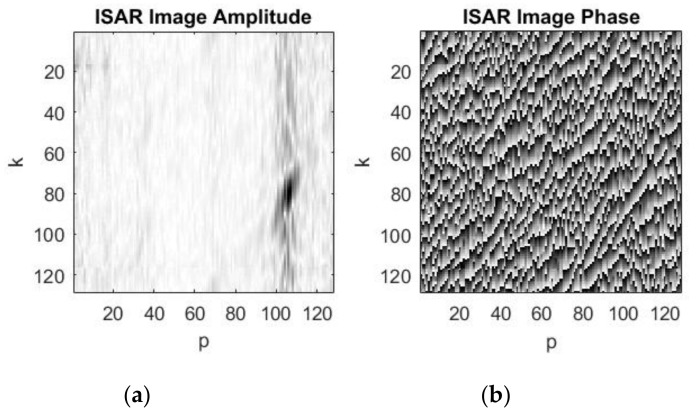
Resulting complex image after 10th additive summation of complex images: (**a**) ISAR image amplitude; (**b**) ISAR image phase.

A standard additive coherent summation (overlaying) of consecutive complex images is applied to reduce the level of the additive white Gaussian noise. The process of the noise depression and image quality improving by additive coherent summations of 3, 8, and 10 complex images is illustrated in Figure 9, Figure 10 and Figure 11, respectively.

The final complex image, amplitude and phase, obtained after additive Gaussian noise depression by coherent summation of 10 consecutive complex images of the asteroid is presented in Figure 11a,b. As can be seen, the asteroid’s ISAR amplitude image is of satisfactory quality, but noise still remains, the asteroid’s silhouette is satisfactorily depicted. Further improving of the ISAR image quality can be achieved by increasing the number of coherent summations which is limited by a huge processing time and graphical properties of the software on which the experiment is carried out.

## 8. Conclusions

In the present work, on the base of real astrophysical measurements by radio telescopes Goldstone-Apple Valley and Arecibo an analytical description of the pulsar Crab emission is suggested. The structure of the Crab pulsar emission has been interpreted as multiple monochromatic Gaussian pulses, distributed in a time-frequency signal grid. Models of pulsar signals reflected from the space object are presented as a time delay copy of time frequency distributed monochromatic Gaussian signals. A new range compression technique is applied to the ISAR complex signal through summation of time recorded ISAR signals in all frequency channels. In case the registration spectral resolution is not satisfactory, which limits the unambiguous time interval of coherency, interpolation in the frequency domain is required.

In the present work only one microburst was applied for aperture synthesis. The author states all microbursts can be used for inverse aperture synthesis in order to improve the image quality through a superposition of all images obtained for each microburst inside the main pulse. In addition, a new ISAR procedure can be developed based on the whole structure of the main pulse. It is supposed that it will further improve the quality of the ISAR image. Based on the sparsity of the ISAR signal due to the limited number of frequency channels in which the ISAR signal is registered, a compressed sensing approach, as *l*_0_ and *l*_1_ norm minimization, can be applied in restoring the pulsar signal structure and ISAR image reconstruction.

Future research works will be focused on other properties and applications of pulsar emissions in the area of stellar navigation and early warning systems for asteroid detection and imaging. From a theoretical point of view, new mathematical structures of pulsar signal models and space object’s imaging algorithms based on the atomic clock’s stability and wide bandwidth of pulsars’ emissions will be developed. From a practical point of view, this work will motivate the development of new highly sensitive technologies to detect pulsars reemissions of asteroids and other nonidentified objects. The usage of steady and stable pulsar emissions for object navigation and imaging purposes is not limited by time and space which is the main advantage of this kind of stellar technology. The problem is the weakness of pulsar emissions, especially reemissions by asteroids and their reliable detection, which requires the development of highly sensitive sensors. From an astronomical point of view, it is very tough to detect the asteroid in the space by the narrow antenna beam of the radio telescope that is not able to cover the whole visible space. A network of multiple synchronized radio-telescopes located on the Earth’s surface and directed on different parts of space, or giant phase arrays antennas scanning the space, are needed in order increase the asteroid’s detectability.

## Figures and Tables

**Table 1:** Enhancement of the SNR with the number of imaging segments.

*n*	10^2^	10^3^	10^4^	10^5^	10^6^
10.log_10_ (signal to noise ratio (SNR))	−119	−96	−73	−50	−27
